# Doctor’s Fees

**Published:** 1887-02

**Authors:** 


### DOCTOR'S FEES.

Of late years it would seem that there is less uniformity in doctor's fees than formerly. The doctors in every village, and even in the country, ought to organize medical societies. and get together at stated intervals to interchange views and consult in regard to their mutual interests. Instead of envyings and jealousies, as too often prevail, they should cultivate a kindly and social feeling for each other, and adopt such measures as will not only mutually advance their professional knowledge but will also promote their financial interest. It is a great mistake to suppose that anyone can succeed, permanently at least, by cultivating those petty jealousies which can see and say nothing good of a professional brother. It were far better to pull together, and work for each other in every honorable way. As to charges, they should endeavor, as far as practicable, to make them uniform. Fee bills should be drawn up and each practitioner should agree to conform to the standard—especially should this be done in obstetric cases and in surgical operations. The fees, of course, must vary in different localities. In villages they are usually somewhat larger than in the country, and in cities they are larger than in villages. The expense of living, rents, etc., cause variation in prices in this as in other matters.

The following charges for the ordinary items may be said to constitute an average for the villages of Georgia, which in the cities are from 50 to 100 per cent. higher :

| | |
|---|---|
| For a single visit in the day, and prescription | $1 50 |
| For a single visit at night, and prescription | 2 50 |
| For two or more visits per day, each | 1 00 |
| For Consultation | $5 00 to 10 00 |
| For case of Obstetrics (normal) | 10 00 |
| For after-attention (four or five calls) | 5 00 |
| Far Instrumental delivery | 25 00 to 50 00 |
| For dressing fractured arm | 10 00 |
| For dressing fractured tibia | 20 00 |
| For dressing fractured femur | 20 00 to 50 00 |

The after-attention is charged according to time and trouble.

Amputations are rated about the same as fractures.

